# Do mothers treat children who are similar to them better? The relation between maternal–adolescent neuroticism congruence and a punitive parenting style

**DOI:** 10.3389/fpsyg.2022.934783

**Published:** 2022-12-19

**Authors:** Shun Peng, Lei Xu, Jiwen Chen, Shuangshuang Cai

**Affiliations:** School of Education, Jianghan University, Wuhan, Hubei, China

**Keywords:** neuroticism, parenting style, maternal–adolescent relationship, congruence, adolescent

## Abstract

Parenting style is the relatively stable behaviors parents display during the childrearing process. It is an important factor in children’s socialization and the mother–child relationship. The present study aimed to test the relationship between maternal–adolescent neuroticism congruence and a punitive parenting style. A total of 882 Chinese adolescents and their mothers participated in this study. The results showed that maternal–adolescent high-neuroticism congruence was associated with a more punitive parenting style than low-neuroticism congruence. The more incongruent the maternal–adolescent neuroticism was, the less punitive the parenting style. There were moderating effects of adolescent gender on maternal–adolescent neuroticism congruence/incongruence and punitive parenting style. These findings provide a new perspective for exploring the relationship between parent–child interaction and parenting styles.

## Introduction

Parenting style is the relatively stable behaviors parents display during the childrearing process (Durbin et al., 1993). Yue (1993) made a Chinese revision based on Egna Minnen AV Barndoms Uppfostran compiled by Perris et al. (1980), dividing the parenting styles of mothers into emotional warmth/understanding, overinvolvement, overprotection, punitive/severity, rejection/denial, and favoritism. A punitive parenting style is characterized by rigid rules, verbal and physical hostility, and high penalization of errors, among other features (Yue, 1993). Researchers often view the punitive parenting style as a negative way of parenting (Lei et al., 2018; Khalid et al., 2019). This is because a punitive parenting style has been identified as a risk factor for the development of internalizing and externalizing problems in childhood (Zubizarreta et al., 2019). Compared with Western parents, Chinese parents are less likely to show outward affection and verbal expressions of love (Deater-Deckard et al., 2011) and endorse higher levels of physical punishment and harsh discipline (Wu et al., 2002; Zhang et al., 2017). Therefore, this study used the level of punitive parenting style as a better indicator of parental satisfaction with adolescents.

In recent years, there has been increasing interest in parents’ personalities and parenting styles (Prinzie et al., 2009; Puff and Renk, 2016), including the relationship between parental neuroticism and parenting style (De Haan et al., 2009). Neuroticism has a persistent effect on individual psychology and behavior (Costa and McCrae, 1988). People with higher neuroticism are more likely to have negative psychological experiences and behavioral responses, such as psychological inflexibility, emotion dysregulation, and aggressive behavior (Barlett and Anderson, 2012; Paulus et al., 2016). Neuroticism, the opposite of emotional stability, has received the most attention (Huver et al., 2010), possibly because neuroticism is thought to be more predictive than other personality dimensions (Belsky et al., 1995). Studies have shown that mothers with higher neuroticism are more likely to adopt overprotective and punitive parenting styles (Coplan et al., 2009; Paulus et al., 2016). Conversely, mothers with low neuroticism and shyness anxiety are more likely to adopt supportive and warm parenting styles (Prinzie et al., 2009; Root et al., 2016). However, mothers do not live in a vacuum. The contributions to maternal parenting style might derive not only from the mother but also from the child’s temperament (Bi et al., 2018). For example, the research of Gölcük and Berument (2021) has shown that the coercive parenting style can be predicted by the interaction of maternal extraversion and child persistence.

**FIGURE 1:**
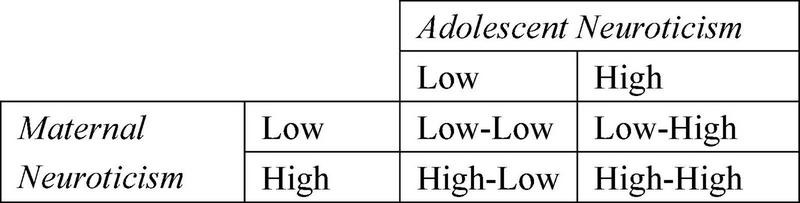
Maternal neuroticism and adolescent neuroticism combination type.

Although researchers have recognized the importance of mother–adolescent interactions and have begun to use dyadic, relational methods to examine the relationship between maternal personality and parenting style (Clark et al., 2000; Aceves and Cookston, 2007; Chaplin et al., 2012; Gölcük and Berument, 2021), previous studies have not been able to explain how a mother with low neuroticism could have a child characterized by high shyness or why maternal neuroticism interacts with child shyness but not with other temperamental characteristics (Coplan et al., 2009; Gölcük and Berument, 2021). There are similarities between the personalities of parents and offspring. For example, the study of Vukasović and Bratko (2015) showed that the heritability of neuroticism ranges from 11 to 24%. From an evolutionary perspective, survival and reproduction are the basic motivations of human beings. To reproduce their genes, individuals invest in their offspring and exhibit an affinity for altruism (Trivers, 1974). According to this view, parents should prefer children who are similar to them in appearance, behavior, and personality (Yu Q. et al., 2019). One way to conceptualize the joint contributions of child and maternal factors to parenting style is within the theoretical framework of “goodness-of-fit” (Chess and Thomas, 1977). Thus, in this study, we focus on the joint effect of maternal and adolescent neuroticism on a maternal punitive parenting style. Due to differences in the degree of maternal neuroticism and adolescent neuroticism, there are four conditions, as shown in Figure 1: high–high, low–low, high–low, and low–high. Based on the earlier conditions, this study aimed to investigate the following: first, when maternal neuroticism is congruent with adolescence, that is, in low–low and high–high conditions, does the punitive parenting style increase with neuroticism? Second, do the punitive parenting style levels of “low–high” and “high–low” conditions differ when maternal neuroticism is incongruent with an adolescent? Third, is the relationship between congruence/incongruence of mother–adolescent neuroticism and punitive parenting style moderated by the gender of the adolescent?

## Literature review

### Neuroticism congruence between mothers and adolescents and punitive parenting style

In recent years, theoretical models of the development of parenting style have shifted away from assigning unique importance to parent or child characteristics toward bidirectional and interactive approaches that take into account both of these influences (Collins et al., 2000; Lengua and Kovacs, 2005; Bornstein et al., 2011). On the one hand, mothers with higher neuroticism tend to be easily distressed, exhibiting nervousness and emotional dysregulation (Barlett and Anderson, 2012; Paulus et al., 2016). This proneness toward negative emotionality might undermine parents’ ability to initiate and maintain positive affective interactions with their children and might limit parents’ ability and willingness to respond adequately to a child’s signals (Prinzie et al., 2009). Mothers with high neuroticism might be more likely to attribute negative intentions to their children when they misbehave, which might result in a negative parenting style (e.g., punitive parenting style) (Coplan et al., 2009; Puff and Renk, 2016). On the other hand, emotionally dysregulated or neurotic children tend to be easily upset and frustrated and difficult to soothe (Rubin et al., 1995). Such children are thought to evoke hostile, critical, punitive, and other authoritarian responses from parents (Lerner et al., 1998). Conversely, mothers with low neuroticism or high emotional stability are calm and self-satisfied (Coplan et al., 2009). They were less likely to adopt a negative parenting style (Prinzie et al., 2009). In addition, adolescents exhibiting low neuroticism are more emotionally stable, making it easier for their mothers to cope and thus reducing the adoption of negative parenting styles (Kochanska et al., 2012). Therefore, this study hypothesized that when mother–adolescent neuroticism is congruent, there is a higher punitive parenting style level in the “high–high” condition than in the “low–low” condition (H1).

### Neuroticism incongruence between mothers and adolescents and punitive parenting style

However, adolescents’ personalities are influenced not only by their mothers but also by other factors (e.g., fathers’ parenting style and genes) (De Haan et al., 2009). Thus, maternal–adolescent neuroticism might not always be congruent. When it is incongruent, it is also necessary to distinguish between “high–low” (high maternal neuroticism and low adolescent neuroticism) and “low–high” (low maternal neuroticism and high adolescent neuroticism) conditions. When mothers exhibit high neuroticism and adolescents low neuroticism, the mother is better able to cope because the adolescents’ emotions are more stable, and an emotionally stable child can better pacify a mother’s negative emotions, which leads to a less negative parenting style (e.g., a less punitive parenting style) (Paulus et al., 2016). Similarly, when adolescents have high neuroticism and mothers have low neuroticism, even though these adolescents are easily upset, frustrated, and difficult to soothe (Rudy and Grusec, 2006), emotionally stable mothers have more patience with them and adopt less negative parenting styles (e.g., punitive parenting styles). Thus, the more incongruent maternal–adolescent neuroticism is, the less punitive the parenting style adopted by mothers.

Specifically, according to Prinzie et al. (2009), more neurotic and less agreeable parents are more likely to attribute negative intentions to their young children when they misbehave and are less likely to provide autonomous support (Van Der Kaap-Deeder et al., 2019). Conversely, parents who score higher on agreeableness and lower on neuroticism are more inclined to tolerate or even support children’s striving toward autonomy, viewing it positively rather than as an attack on parental authority. Therefore, the punitive parenting style level in the “low–high” condition is likely to be lower than that in the “high–low” condition. Therefore, the study hypothesized that when mother–adolescent neuroticism is incongruent, the punitive parenting style level in the “low–high” condition is lower than that in the “high–low” condition (H2). In addition, based on the previous description, when both mothers and adolescents have low neuroticism, the maternal punitive parenting style may be lower. Therefore, we hypothesized that the “low–low” condition has the lowest punitive parenting style (H3).

### Moderation role of gender

Notably, there are also differences in how mothers treat their children of different genders. According to the social role theory (Wood and Eagly, 2012), boys and girls learn how to be men and women through imitation in childhood. Parents also guide gender learning through things such as clothes, chores, and toys. According to Leaper (2002), studies show that girls need more emotional support and boys need more independence from their parents. Therefore, girls need more parental acceptance, and boys need more parental autonomy granting. Moreover, Chodrow (1978) presented a psychoanalytical view of gender differences in parenting. According to this theory, because the mother and daughter are of the same sex, the daughter’s identification with the mother is much stronger than that of the son. Even in high–high conditions, we still need to consider differences in the punitive parenting style between mother–son and mother–daughter relationships. In other words, when in the high–high condition, because mothers have a stronger bond with their daughters than with boys, if a mother always imposes punishment, it may cause a girl to “pull away” from the mother, thus reducing the bond. To maintain this bond, mothers may lower the punitive parenting style with girls. In contrast, due to the higher expectations of sons in traditional Chinese culture, when boys do not conform to their parents’ expectations of behavior and performance (such as emotional instability), parents will punish them with the idea of “spare the rod spoil the son.” That is, when the mother’s and son’s neuroticism level is the high–high condition, the neuroticism level increases, the punitive parenting style increases. Therefore, this study hypothesized that when mother–adolescent neuroticism is in the high–high condition, the punitive parenting style levels in the mother–daughter group increase with the neuroticism level and then decrease after neuroticism reaches a certain level. However, in the mother–boy group, the punitive parenting style increases linearly with the increasing neuroticism level (H4).

Similarly, according to Prinzie et al. (2009), highly neurotic mothers were more likely to attribute negative intentions to their children, regardless of their sex. In contrast, mothers with low neuroticism are more likely to tolerate their children and give them more autonomous support (Van Der Kaap-Deeder et al., 2019). Therefore, this study hypothesized that when mother–adolescent neuroticism is incongruent, the maternal punitive parenting style levels in the “low–high” condition are lower than those in the “high–low” condition, regardless of the sex of the child (H5).

In summary, studies have shown similarities between maternal and adolescent personalities (Vukasović and Bratko, 2015; Yu Q. et al., 2019). However, the effect of maternal–adolescent personality congruence on maternal parenting style and the moderating role of adolescent gender are less known. The current study focused on the relationship between maternal–adolescent neuroticism congruence and a punitive parenting style and the moderating role of adolescent gender.

## Materials and methods

### Participants and procedures

The university Research Ethics Committee approved the present study. After obtaining informed consent from the schools, 18 classes were selected from three middle schools in mainland China (six classes from three grades were randomly selected), for a total of 934 pair participants. All participants signed informed consent forms. A total of 882 adolescents and their mothers completed the questionnaires, for a 94.43% retention rate. Among the retained adolescent participants, 423 were male participants (48.00%) and 459 were female participants (52.00%). A total of 302 were in grade 7 (34.24%; 143 male participants, 159 female participants), 290 were in grade 8 (32.88%; 139 male participants, 151 female participants), and 290 were in grade 9 (32.88%; 141 male participants, 149 female participants). The mean age of the participants was 13.99 years (SD = 0.82). There was no significant gender difference by grade for the adolescent participants [χ^2^(2) = 0.10, *p* > 0.05]. The adolescent participants were given a small gift after completing the questionnaire. The mean age of the mothers was 38.15 years (SD = 1.25).

### Measures

#### Maternal neuroticism

Maternal neuroticism was measured by the neuroticism subscale of the brief version of the Chinese Big Five Personality Inventory (CBF-PI-B) (Wang et al., 2011). The subscale includes eight items (e.g., “Sometimes I feel worthless”). Responses are given on a six-point Likert-type scale (from 1 = strongly disagree to 6 = strongly agree). The higher the score is, the greater the neuroticism. The Cronbach’s α of the subscale was 0.81 in this study.

#### Adolescent neuroticism

Adolescent neuroticism was assessed with the neuroticism subscale of the brief version of the Chinese Big Five Personality Inventory (CBF-PI-B) (Wang et al., 2011). The subscale includes eight items (e.g., “I often feel insecure inside”). Responses are given on a six-point Likert-type scale (from 1 = strongly disagree to 6 = strongly agree). The higher the score is, the greater the neuroticism. In the present study, the Cronbach’s α of the subscale was 0.87.

#### Punitive parenting style

We used the maternal punitive parenting style subscale of the Chinese version of the Egna Minnen av Barndoms Uppfostran (EMBU) to assess punitive parenting style (Yue, 1993). The subscale includes nine items (e.g., “My mother often punished me more than I deserved”). Participants were asked to indicate their agreement on a four-point scale (from 1 = never to 4 = always). The higher the score is, the more punitive the parenting style as perceived by the adolescent. In the present study, the Cronbach’s α of the subscale was 0.88.

### Data analysis

First, to test the effect of maternal–adolescent neuroticism congruence or incongruence on punitive parenting style, we use quadratic polynomial regression (Edwards and Parry, 1993). According to Edwards and Parry (1993) procedure, we can use Equation 1 to test the congruence/incongruence effect of maternal neuroticism (*X*) and adolescent neuroticism (*Y*) on punitive parenting style (*Z*).


(1) $$Z=b_{0}+b_{1}X+b_{2}Y+b_{3}X^{2}+b_{4}XY+b_{5}Y^{2}+e$$

Then, we test the slopes and curvatures of the congruence line (*X = Y*) and the incongruence line (*X = –Y*) to explain the relationship between maternal–adolescent neuroticism congruence/incongruence and a punitive parenting style. However, the coefficients of quadratic polynomial regression are hard to explain. Edwards and Parry (1993) suggested using polynomial regression results to generate a three-dimensional response surface to test the effect of congruence on the dependent variables (Edwards and Parry, 1993). According to Edwards and Parry (1993), a significant congruence effect exists when the coefficients for the three second-order polynomial terms (i.e., *X*^2^, *XY*, and *Y*^2^) are jointly significant and the curvature along the incongruence line is significantly different from zero (Edwards and Parry, 1993). In addition, when the slope along the congruence line (*X = Y*) is significant and positive, we can conclude that congruence at high levels of neuroticism results in more pronounced outcomes than congruence at low levels.

In addition, to test the moderating effect of gender, gender (*W*) is added to Equation 1 according to the moderated regression model (e.g., Aiken et al., 1991), and the following equation is obtained:


(2) $$\begin{array}{l} Z=b_{0}+b_{1}X+b_{2}Y+b_{3}X^{2}+b_{4}XY+b_{5}Y^{2}+b_{6}W\\ \;\;+b_{7}WX+b_{8}WY+b_{9}WX^{2}+b_{10}WXY\\ \;\;+b_{11}WY^{2}+e \end{array}$$

The moderating effect of *W* is captured by the five terms *WX*, *WY*, *WX*^2^, *WXY*, and *WY*^2^ as a set. Moderation is tested by assessing the increment in *R*^2^ yielded by the terms *WX*, *WY*, *WX*^2^, *WXY*, and *WY*^2^, which amounts to testing whether the *R*^2^ from Equation 2 is larger than the *R*^2^ from Equation 1.

The simple quadratic functions indicated by Equation 3 can be derived by substituting selected values of W. For instance, assume *W* is a dichotomous variable representing gender, in which *W* = 0 for boys and *W* = 1 for girls. The equation for boys is derived by substituting *W* = 0 into Equation 2, which yields:


(3) $$Z=b_{0}+b_{1}X+b_{2}Y+b_{3}X^{2}+b_{4}XY+b_{5}Y^{2}+e$$

Note that Equation 3 reduces to Equation 1, given that each term involving *W* becomes zero and therefore drops out. The equation for girls is found by substituting *W* = 1 into Equation 2, which produces:


(4) $$\begin{array}{l} Z=\left(b_{0}+b_{6}\right)+\left(b_{1}+b_{7}\right)X+\left(b_{2}+b_{8}\right)Y+\left(b_{3}+b_{9}\right)X^{2}\\ \;\;+\left(b_{4}+b_{10}\right)XY+\left(b_{5}+b_{11}\right)y^{2}+e \end{array}$$

In this study, *R* was used to analyze the data, the rstatix package was used to conduct descriptive statistics, the lavaan package was used for testing polynomial regression, and the RSA package was used for visualizing the response surface.

## Results

### Preliminary analysis

The results of the preliminary analysis (see Table 1) showed that maternal neuroticism was positively correlated with adolescent neuroticism and a punitive parenting style. Adolescent neuroticism was positively correlated with a punitive parenting style.

**TABLE 1:** Mean, standard deviation, and correlation coefficient of each variable.

Variables	*M*	SD	1	2	3	4
1. Age	13.99	0.82	–			
2. Gender*	0.52	0.50	–	–		
3. Maternal neuroticism	2.46	0.89	0.01	−0.08*	–	
4. Adolescent neuroticism	3.21	1.19	–0.02	0.12***	0.15***	–
5. Punitive parenting style	1.45	0.52	–0.03	0.16***	0.18***	0.35***

*n* = 882. *p < 0.05, ***p < 0.001. Gender* was coded 0 = females and 1 = males.

### The relationship between neuroticism congruence/incongruence and a punitive parenting style

**FIGURE 2:**
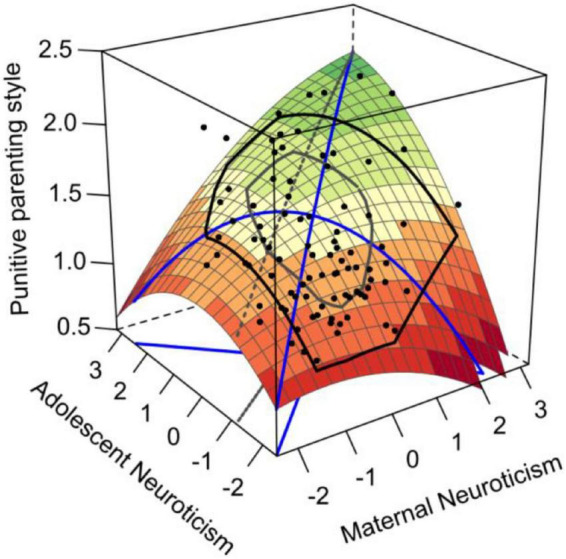
Congruence effect and asymmetrical incongruence effect of maternal–adolescent neuroticism on punitive parenting style.

We used polynomial regression to test the effects of maternal neuroticism and adolescent neuroticism congruence on punitive parenting style. Table 2 shows the regression coefficients as well as the slopes and curvatures of the maternal neuroticism and adolescent neuroticism congruence/incongruence lines. The response surface plot is generated based on these coefficients. In Figure 2, the congruence line (MN = AN) runs from the left corner (where MN = AN = –2) to the right corner (where MN = AN = 3), whereas the incongruence line (MN = –AN) runs from the front corner to the rear corner. According to Figure 2 and Table 2, the congruence line shows a monotonic increase (*a*_1_ = 0.25, *p* < 0.001; *a*_2_ = –0.02, *p* > 0.05), indicating that the maternal–adolescent high–high neuroticism congruence condition is correlated with a more punitive parenting style than the low–low neuroticism congruence condition; thus, hypothesis 1 was supported.

**TABLE 2:** Polynomial regressions of punitive parenting style on neuroticism congruence/incongruence.

Variables		Punitive parenting style			
		
		*B*	SE	ci.lower	ci.upper
Constant (*b*_0_)		1.54***	0.25	1.49	1.59
Maternal neuroticism (MN) (*b*_1_)		0.08**	0.02	0.04	0.11
Adolescent neuroticism (AN) (*b*_2_)		0.18***	0.01	0.15	0.20
MN^2^ (*b*_3_)		−0.04*	0.01	–0.06	–0.01
MN × AN (*b*_4_)		0.07**	0.02	0.04	0.10
AN^2^ (*b*_5_)		−0.06**	0.01	–0.07	–0.04
		*R*^2^ = 0.18, *p* < 0.001			

**Congruence/Incongruence**	**Slope/Curvature**	**Effect**	**SE**	**ci.lower**	**ci.upper**

Congruence line (MN = AN)	*a*_1_ = *b*_1_ + *b*_2_	0.258***	0.02	0.21	0.29
	*a*_2_ = *b*_3_ + *b*_4_ + *b*_5_	–0.02	0.02	–0.05	0.01
Incongruence line (MN = –AN)	*a*_3_ = *b*_1_ – *b*_2_	−0.10**	0.02	–0.14	–0.05
	*a*_4_ = *b*_3_ – *b*_4_ + *b*_5_	−0.16***	0.03	–0.21	–0.11
	*a*_5_ = *b*_3_ – *b*_5_	0.02	0.01	–0.01	0.04

*n* = 882. Unstandardized coefficients are reported. **p < 0.01 and ***p < 0.001. Maternal neuroticism (MN) and adolescent neuroticism (AN) were centered before calculating the second-order terms.

The surface along the incongruence line curves downward (*a*_4_ = –0.16, *p* < 0.001). The slope on the incongruent line was significantly negative (a*_3_* = –0.10, *p* < 0.001), indicating that the punitive parenting style in the low–high condition was higher than that in the high–low condition. H2 was not supported.

To test hypothesis 3, we followed Edwards procedure^1^ : we first centralized the data and then set the value to –2 (low) or 2 (high) for maternal and adolescent neuroticism, respectively. The results showed that in the low–low condition, the punitive parenting style was significantly higher than in the high–low condition, with a value of 0.27 (*p* = 0.02). H3 was not supported.

### Test the moderating role of adolescent gender

**FIGURE 3:**
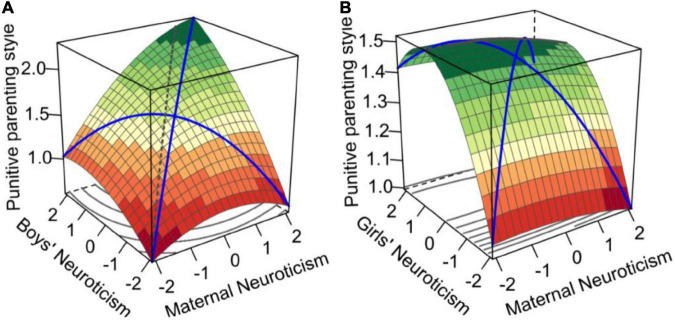
Congruence effect and asymmetrical incongruence effect of maternal–adolescent neuroticism on punitive parenting style in different genders **(A)** for boys and **(B)** for girls.

We used moderated polynomial regression to test the moderation effects of gender between maternal neuroticism and adolescent neuroticism congruence on punitive parenting style. Table 3 and Figure 3 shows the slopes and curvatures of the maternal neuroticism and adolescent neuroticism congruence/incongruence lines. The response surface plot is generated based on these coefficients (see Table 4). The Δ*R*^2^ of Model 2 and Model 1 was 0.065, *p* < 0.001, indicating that adolescent gender moderated the relationship between maternal–adolescent neuroticism congruence/incongruence and punitive parenting style.

**TABLE 3:** Gender difference of congruence effect on punitive parenting style.

Gender	Congruence/Incongruence	Slope/Curvature	Effect	SE	ci.lower	ci.upper
*Daughter*	Congruence line (MN = AN)	*a*_11_	0.07*	0.03	0.01	0.13
		*a*_21_	−0.07*	0.02	–0.11	–0.02
	Incongruence line (MN = –AN)	*a*_31_	−0.10**	0.03	–0.15	–0.04
		*a*_41_	−0.06*	0.03	–0.13	–0.004
*Son*	Congruence line (MN = AN)	*a*_12_	0.40***	0.03	0.34	0.47
		*a*_22_	–0.03	0.04	–0.11	0.05
	Incongruence line (MN = –AN)	*a*_32_	−0.09**	0.04	–0.19	–0.01
		*a*_42_	−0.17***	0.06	–0.30	–0.05

*p < 0.05, **p < 0.01 and ***p < 0.001.

**TABLE 4:** Moderated polynomial regression result.

Variables	Model 1 (DV*:* Punitive parenting style)				Model 2 (DV*:* Punitive parenting style)			
		
	*B*	SE	ci.lower	ci.upper	*B*	SE	ci.lower	ci.upper
Constant (*b*_0_)	1.54***	0.25	1.49	1.59	1.47***	0.03	1.41	1.54
Maternal neuroticism (MN) (*b*_1_)	0.08**	0.02	0.04	0.11	–0.02	0.03	–0.06	0.03
Adolescent neuroticism (AN) (*b*_2_)	0.18***	0.01	0.15	0.20	0.09**	0.02	0.05	0.11
MN^2^ (*b*_3_)	−0.04*	0.01	–0.06	–0.01	–0.01	0.01	–0.03	0.02
MN × AN (*b*_4_)	0.07**	0.02	0.04	0.10	–0.002	0.02	–0.05	0.04
AN^2^ (*b*_5_)	−0.06*	0.01	–0.07	–0.04	−0.06*	0.01	–0.08	–0.04
Gender (*b*_6_)					0.10	0.05	–0.01	0.20
Gender × MN (*b*_7_)					0.17***	0.04	0.09	0.25
Gender × AN (*b*_8_)					0.16***	0.03	0.11	0.22
Gender × MN^2^ (*b*_9_)					–0.05	0.04	–0.13	0.03
Gender × MN × AN (*b*_10_)					0.07	0.04	–0.004	0.15
Gender × AN^2^ (*b*_11_)					0.01	0.02	–0.02	0.05
	*R*^2^ = 0.18				*R*^2^ = 0.24, Δ*R*^2^ = 0.065, *p* < 0.001			

*n* = 882. Unstandardized coefficients are reported. *p < 0.05, **p < 0.01 and ***p < 0.001. MN and AN were centered before calculating the second-order terms and third-order terms.

DV, dependent variable.

According to Table 3, *in the mother–daughter group*, the congruence line (MN = AN) shows an invest U-shaped relationship (*a*_11_ = 0.07, *p* < 0.05; *a*_21_ = –0.07, *p* < 0.05), indicating that when the neuroticism of mothers and girls was congruent, the punitive parenting style increased as the neuroticism levels increased but then decreased as the neuroticism levels reached a certain point. On the incongruent line (MN = –AN), the curvature was significantly negative (*a*_41_ = –0.17, *p* < 0.01), indicating that the punitive parenting style level decreased with increases in the degree of neuroticism incongruence between mothers and girls. In addition, the slope had a significant negative value (*a*_31_ = –0.09, *p* < 0.01), indicating that the punitive parenting style in the low–high condition was higher than that in the high–low condition.

*In the mother–son group*, the congruence line shows a monotonic increase (*a*_12_ = 0.40, *p* < 0.001; *a*_22_ = –0.03, *p* > 0.05), indicating that the maternal–adolescent high–high neuroticism congruence condition is correlated with a more punitive parenting style than the low–low neuroticism congruence condition. On the incongruent line (MN = –AN), the curvature was significantly negative (*a*_42_ = –0.06, *p* < 0.05), indicating that the punitive parenting style level increased with the increase in neuroticism congruity between mothers and boys. The slope had a significant negative value (*a*_32_ = –0.10, *p* < 0.001), indicating that the punitive parenting style in the “low–high” condition was higher than that in the “high–low” condition. Hypothesis 4 was supported, but hypothesis 5 was not supported.

## Discussion

Although the effects of mother–adolescent interactions on parenting style have been investigated, researchers have not yet tested the effects of maternal and adolescent neuroticism similarity/congruence on parenting style. In this study, a complex set of inter associations was observed among adolescent neuroticism, punitive parenting style, and maternal neuroticism. In fact, maternal neuroticism can be passed on to children (Vukasović and Bratko, 2015). In addition, mothers and adolescents are influenced by the same family environment (Yu Q. et al., 2019). Both genes and the environment provide the basis for congruence between mothers and adolescents in terms of personality. Therefore, it is necessary to investigate the influence of maternal–adolescent personality congruence/similarity on maternal parenting style.

### Maternal–adolescent neuroticism congruence/incongruence and punitive parenting style

One finding of this study was that maternal–adolescent high-neuroticism congruence was associated with a more punitive parenting style than low-neuroticism congruence, and hypothesis 1 was supported. Neurotic or anxious mothers tend to overreport their children’s difficult temperament, which might result in a negative parenting style (e.g., a punitive parenting style) (Scalzo et al., 2005; Desjardins et al., 2008). This study provides a new perspective for investigating the relationship between parent–child interaction and parenting styles. The results suggest that the way mothers raise their offspring is not based on a “genetic perspective” but is shaped by real-world interactions. Mothers, indeed, do not necessarily bring up adolescents based on a “genetic perspective.” However, maternal–adolescent neuroticism congruence leads to a more punitive parenting style. This, in turn, may further increase neuroticism in adolescents, leading to the “inheritance” of neuroticism.

The present study showed that the more incongruent maternal–adolescent neuroticism is, the less punitive the parenting style adopted by mothers. Similar results were found across gender groups. Specifically, the punitive parenting style in the low–high condition was higher than that in the high–low condition, H2 and H5 were not supported. This may be because neuroticism in adolescents also “predicts” other risk factors, such as more negative parenting styles of the fathers when adolescent neuroticism is high (Yu J. et al., 2019) or more stressful life events (Otis et al., 2016). In these situations, mothers need more resources to deal with other risk factors (such as negative parenting by the father) and are more likely to adopt a punitive parenting style. In contrast, when adolescent neuroticism is low and maternal neuroticism is high, low adolescent neuroticism may also “predict” protective factors in the domestic environment, where mothers are more likely to cope with their children and thus have lower levels of punitive parenting.

In addition, H3 was not supported. This may be because people have more frequent contact with those similar to them than with those dissimilar to them (McPherson et al., 2001). Meanwhile, parents and children commonly live in the same family, which provides a high baseline level of contact. This stronger bond may result in the mother spending more time with the teen, thus allowing the mother greater “exposure” to the teen’s problem behavior, thus exacerbating the level of punitive parenting style.

### Moderation role of adolescents’ gender

This study verified the moderating effect of adolescents’ gender on the relationship between maternal–adolescent neuroticism congruence/incongruence and punitive parenting style. Specifically, in the mother–girl group, the congruence line (MN = AN) showed an inverted U-shaped relationship, which means that when mothers’ and teenagers’ neuroticism were congruent, the punitive parenting style increased as the neuroticism level increased but then decreased as the neuroticism level reached a certain level. In the mother–son group, the congruence line showed a monotonic increase, indicating that the maternal–adolescent high–high neuroticism congruence condition was correlated with a more punitive parenting style than the low–low neuroticism congruence condition. The results supported previous research, suggesting that parents use different parenting styles when interacting with children of different genders. Investigating the moderating effect of adolescents’ gender is helpful to respond to the relationship between maternal–adolescent neuroticism congruence/incongruence and punitive parenting style. The results revealed in-depth the impact of mother–son interaction and mother–daughter interaction on punitive parenting style.

### Strengths, limitations, and future directions

Several strengths of this study should be noted. First, previous studies have examined the influence of the interaction between mother’s personality and child’s traits on parenting style, but few studies have explored the influence of this similarity on the parental style based on the perspective of “mother–child similarity.” Based on the methods of polynomial regression and response surface analysis, this study deeply discussed the influence of mother–adolescent personality differences on parenting style, which provided new evidence for explaining the generation mechanism of parenting style. Second, although previous studies have realized that parents may have different parenting styles for children of different genders, they have not thoroughly explored the possible consequences of parent–child interaction between children of different genders. This study examined the moderating effect of gender on the relationship between maternal–adolescent neuroticism congruence/incongruence and punitive parenting style. The results suggested that the interaction between mothers and children of different genders affected punitive parenting style.

Limitations also should be considered. First, although our design was guided by established theories and research results, our data are cross-sectional. Investigations that include longitudinal data are needed to verify these relationships in future studies. Second, all the participants in this study were from China, and the specific cultural context (e.g., parents are more likely to adopt an authoritarian parenting style) may limit the generalization of the findings made in the study (Rudy and Grusec, 2006). Future studies could collect data from different cultures to address this limitation. Third, this study examined the punitive parenting styles of mothers, which cannot yet reflect the “complete picture” of parenting styles. Future studies can examine how the interactions between fathers and adolescents impact punitive parenting styles. Fourth, the present study collected data on punitive parenting style, and future studies can investigate the other dimensions of parenting style.

## Conclusion

The present study is the first, to our knowledge, to explore the effect of maternal–adolescent neuroticism congruence on maternal parenting style. Our results showed that maternal–adolescent high-neuroticism congruence was associated with a more punitive parenting style than low-neuroticism congruence. The more incongruent the maternal–adolescent neuroticism is, the less punitive the parenting style. Adolescent gender moderated the relationship between maternal–adolescent neuroticism congruence and punitive parenting style. In practice, this study suggests that not only mothers’ influence but also children play an important role in parenting style. Therefore, when raising children, mothers should pay attention to their own and their children’s behaviors to create a positive and stable family atmosphere, providing a foundation for promoting adolescents’ positive development.

## Data availability statement

The original contributions presented in this study are included in the article/supplementary material, further inquiries can be directed to the corresponding author.

## Ethics statement

The studies involving human participants were reviewed and approved by the Jianghan University Research Ethics Committee. Written informed consent to participate in this study was provided by the participants or their legal guardian/next of kin.

## Author contributions

SP and JC conducted literature review, analyzed the data, and wrote the first draft of the manuscript. LX and SP generated the ideal of the study. SC and SP participated in the data collection and improved the manuscript substantially. All authors contributed to the article and approved the final manuscript.

## Footnotes

1
http://public.kenan-flagler.unc.edu/faculty/edwardsj/ComparingPointsResponseSurface.htm
